# Supplementary material for: FAM3A reshapes VSMC fate specification in abdominal aortic aneurysm by regulating KLF4 ubiquitination
Source: Nat Commun. 2023 Sep 2;14:5360. doi: 10.1038/s41467-023-41177-x (PMC10475135; doi:10.1038/s41467-023-41177-x)
Supplement: Supplementary file 1 — Supplementary information [file 41467_2023_41177_MOESM1_ESM.pdf]

Supplementary Information for

FAM3A reshapes VSMC fate specification in abdominal aortic aneurysm by regulating KLF4 ubiquitination

Chuxiang Lei *et al.*

Correspondence to:
zhengyuehong2022@outlook.com
dyang0226@163.com

This PDF file includes:

- Supplementary Figs. 1 to 12;
- Supplementary Tables 1-7 (animals, cells, and reagent major resources are included in Tables 4-7);
- Supplementary References

Supplementary Figures

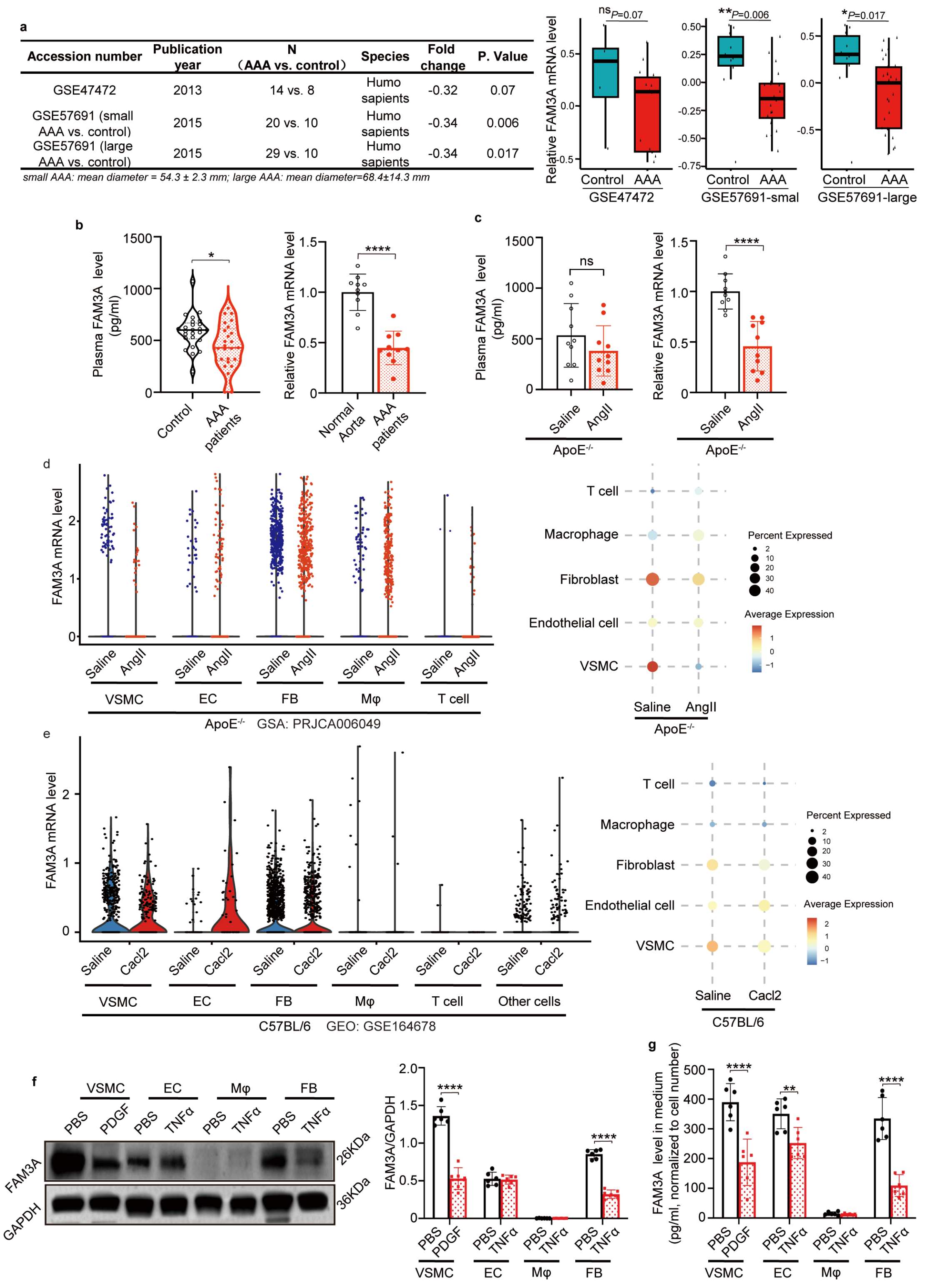

Accession number	Publication year	N (AAA vs. control)	Species	Fold change	P. Value
GSE47472	2013	14 vs. 8	Humo sapients	-0.32	0.07
GSE57691 (small AAA vs. control)	2015	20 vs. 10	Humo sapients	-0.34	0.006
GSE57691 (large AAA vs. control)	2015	29 vs. 10	Humo sapients	-0.34	0.017

small AAA: mean diameter = 54.3 ± 2.3 mm; large AAA: mean diameter=68.4±14.3 mm

Supplementary Fig. 1 FAM3A expression in vivo and in vitro. a Public transcript microarray datasets from the GEO database with AAA patients and normal control subjects were analyzed, and the FAM3A mRNA levels are shown. **b** Quantification of plasma FAM3A protein levels (n=25 biologically independent samples) and mRNA levels in aortas is shown from AAA patients or normal control subjects (n=10 biologically independent samples). **c**

Quantification of plasma FAM3A protein levels (n=10 biologically independent samples) and mRNA levels in aortas (n=10 biologically independent samples) is shown from AngII-ApoE$^{-/-}$ murine AAA models and control models. **d, e** The cell-specific FAM3A mRNA level in aortas were analyzed from single-cell RNA sequencing transcript profiles in AngII-ApoE$^{-/-}$ murine AAA models (n=9,338 cells) versus control models (n=7,914 cells) (GSA, under the code PRJCA006049, d) and in C57BL/6-Cacl2 murie AAA models (n=1,620 cells) versus control models (n=2,801 cells) (GEO, under the code GSE164678, e). The quantification of FAM3A expression levels corresponding to individual cell is shown (left, d and e). The quantification of FAM3A expression levels corresponding to each cell type is shown (the size of circles denotes the contribution fraction of this cell type to the total FAM3A expression in aortas; the color gradation denotes the average FAM3A expression level of individual cell by quantifying the total FAM3A expression within this cell type divided by cell number within the same type; right, d and e). **f, g** The quantification of FAM3A protein levels in the whole cell lysates (f) and in the culture medium (g) in primary human vascular smooth muscle cell (VSMC), endothelial cell (EC), fibroblast (FB), or macrophage (Mφ), respectively, treated with pathological stimuli as indicated in the chart (PDGF-BB, 10 ng/mL for 24 hours; TNFα, 25 ng/mL for 6 hours; each value of FAM3A protein level in the culture medium was normalized to its corresponding cell number in the culture plate, n=6 biologically independent experiments in f, g). Quantitative comparisons between samples were run on the same gel (f). Data are presented as median (+Max, −Min; a) and mean±SEM (b, c, f, g). Statistical significance was calculated with moderated t-test (a), two-tailed independent *t* test (b, c) and two-way ANOVA followed by Tukey post hoc test (f, g) and *P* values are indicated ($^{ns}P\geq0.05$, $*P<0.05$, $**P\leq0.01$, $***P\leq0.001$, $****P\leq0.0001$). Source data are provided as a Source Data file.

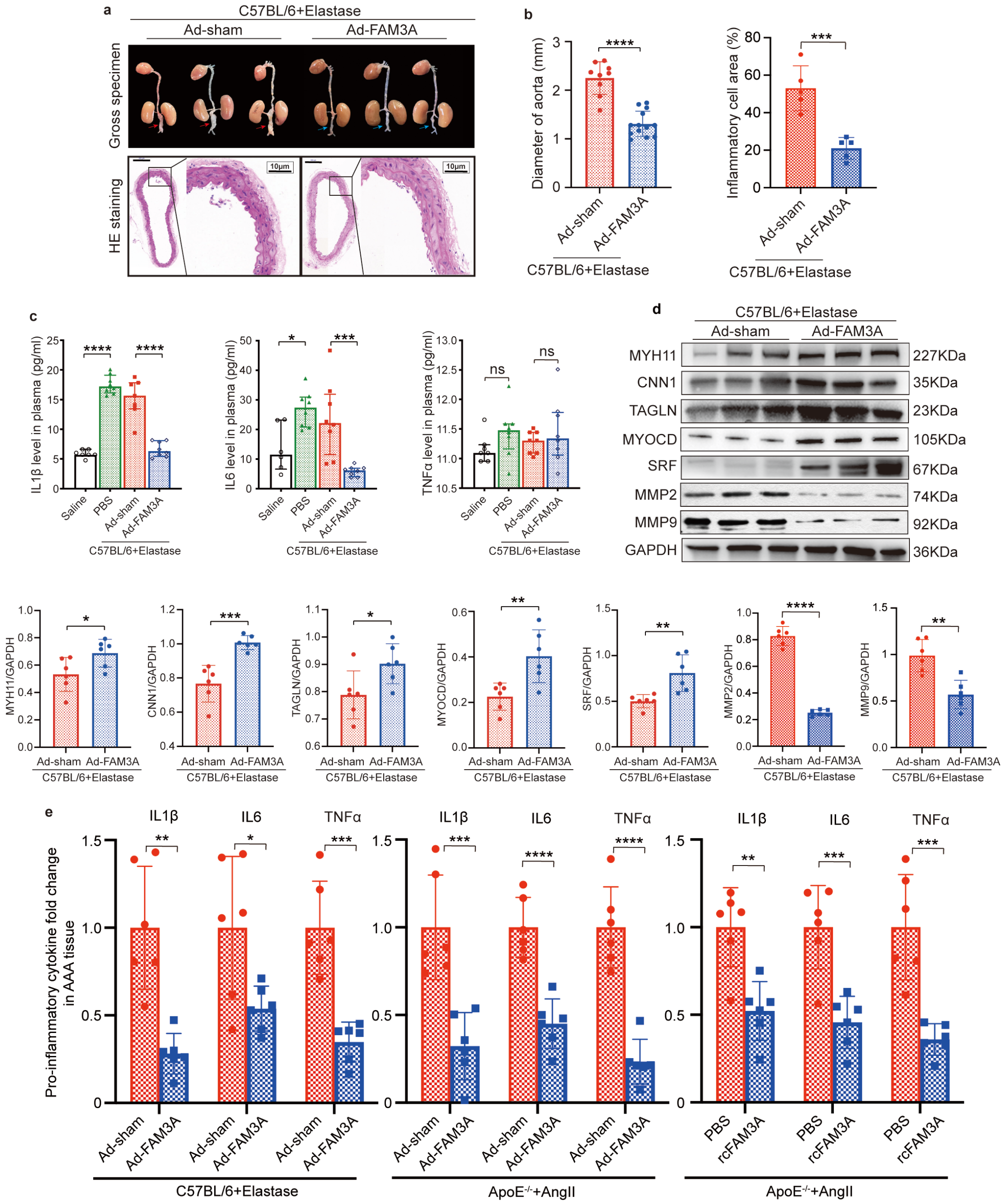

Supplementary Fig. 2 FAM3A overexpression attenuates AAA formation in Elastase-C57BL/6 murine AAA models. a Gross specimen of whole aortas and HE staining of aortic cross section from Elastase-C57BL/6 murine AAA models and control models. (Representative images of n=9 or 13 biologically independent animals in Ad-sham or Ad-FAM3A, respectively). Scale bar: 200 μm, insets: 10 μm. **b** Quantification of aortic diameter (n=9 or 13 biologically independent animals in Ad-sham or Ad-FAM3A, respectively) and inflammatory cell infiltration in aortic tissues is shown (n=5 biologically independent animals). **c** Quantification of the levels of plasma IL1β, IL6, and TNFα in saline-C57BL/6 mice and Elastase-C57BL/6 murine AAA models treated with or without FAM3A overexpression by adenovirus is shown (n=7, 8, 7, or 8 biologically independent animals in Saline control, Elastase control, Elastase Ad-sham, or Elastase Ad-FAM3A,

respectively for IL1β; n=6, 8, 8, or 8 biologically independent animals in Saline control, Elastase control, Elastase Ad-sham, or Elastase Ad-FAM3A, respectively for IL6; n=7, 8, 8, or 8 biologically independent animals in Saline control, Elastase control, Elastase Ad-sham, or Elastase Ad-FAM3A, respectively for TNFα). **d** Representative western blot images and quantification of VSMC contractile marker proteins in aortas are shown from mice treated as in a (n=6 biologically independent animals; quantitative comparisons between samples were run on the same gel). **e** Quantification of proinflammatory cytokines in aorta from indicated murine AAA models treated with adenovirus-mediated FAM3A overexpression or recombinant FAM3A is shown (n=6 biologically independent animals). Data are presented as mean±SEM (b-e). Statistical significance was calculated with two-tailed independent *t* test (b, d, e) and one-way ANOVA followed by Tukey post hoc test (c) and *P* values are indicated ($^{ns}P \geq 0.\ 05$, $*P < 0.\ 05$, $**P \leq 0.\ 01$, $***P \leq 0.\ 001$, $****P \leq 0.\ 0001$). Source data are provided as a Source Data file.

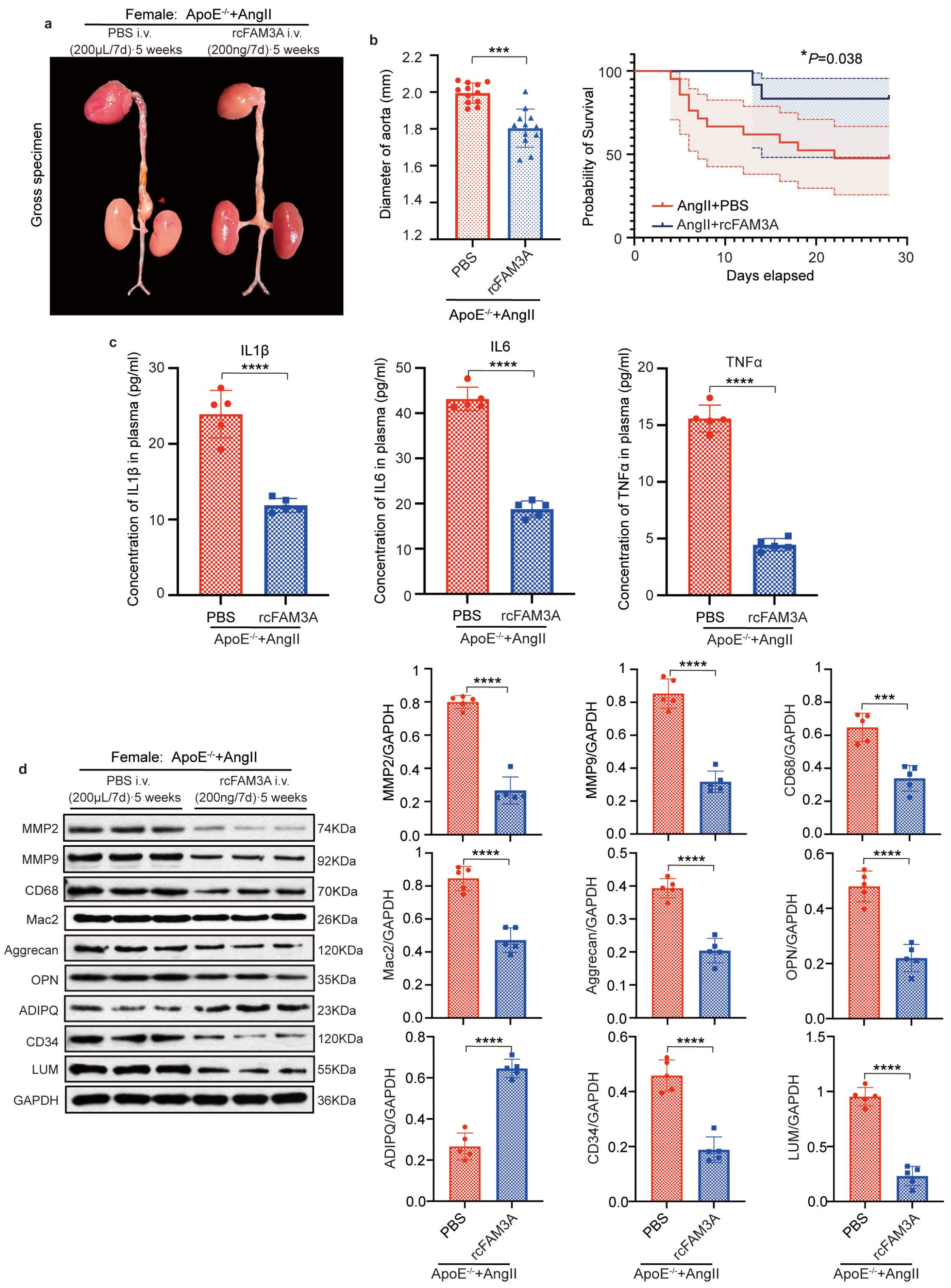

Supplementary Fig. 3 Protective roles of recombinant FAM3A in female AngII-ApoE$^{-/-}$ murine AAA models. **a** Gross specimen images of aortas from female AngII-ApoE$^{-/-}$ murine AAA models treated with or without recombinant FAM3A (Representative images of n=12 biologically independent animals). **b** Quantification of diameters of abdominal aortas and the survival probabilities by Kaplan-Meier curve and log-rank test is shown from mice treated as in a (n=12 biologically independent animals). **c** ELISAs were performed to evaluate the plasma inflammatory factors from mice treated as in a (n=5 biologically independent animals).

d Representative western blot images and quantification of MMPs and VSMC contractile marker proteins are shown in aortas from mice treated as in a (n=5 biologically independent animals; quantitative comparisons between samples were run on the same gel). Data are presented as mean±SEM (Diameter of aorta in b, c, d) and mean±95% CI (Probability of survival in b). Statistical significance was calculated with two-tailed independent *t* test (Diamer of aorta in b, c, d) and Kaplan-Meier analysis and log-rank test (Probability of survival in b) . and *P* values are indicated (**P*<0. 05, ***P*≤0. 01, ****P*≤0. 001, *****P*≤0. 0001). Source data are provided as a Source Data file.

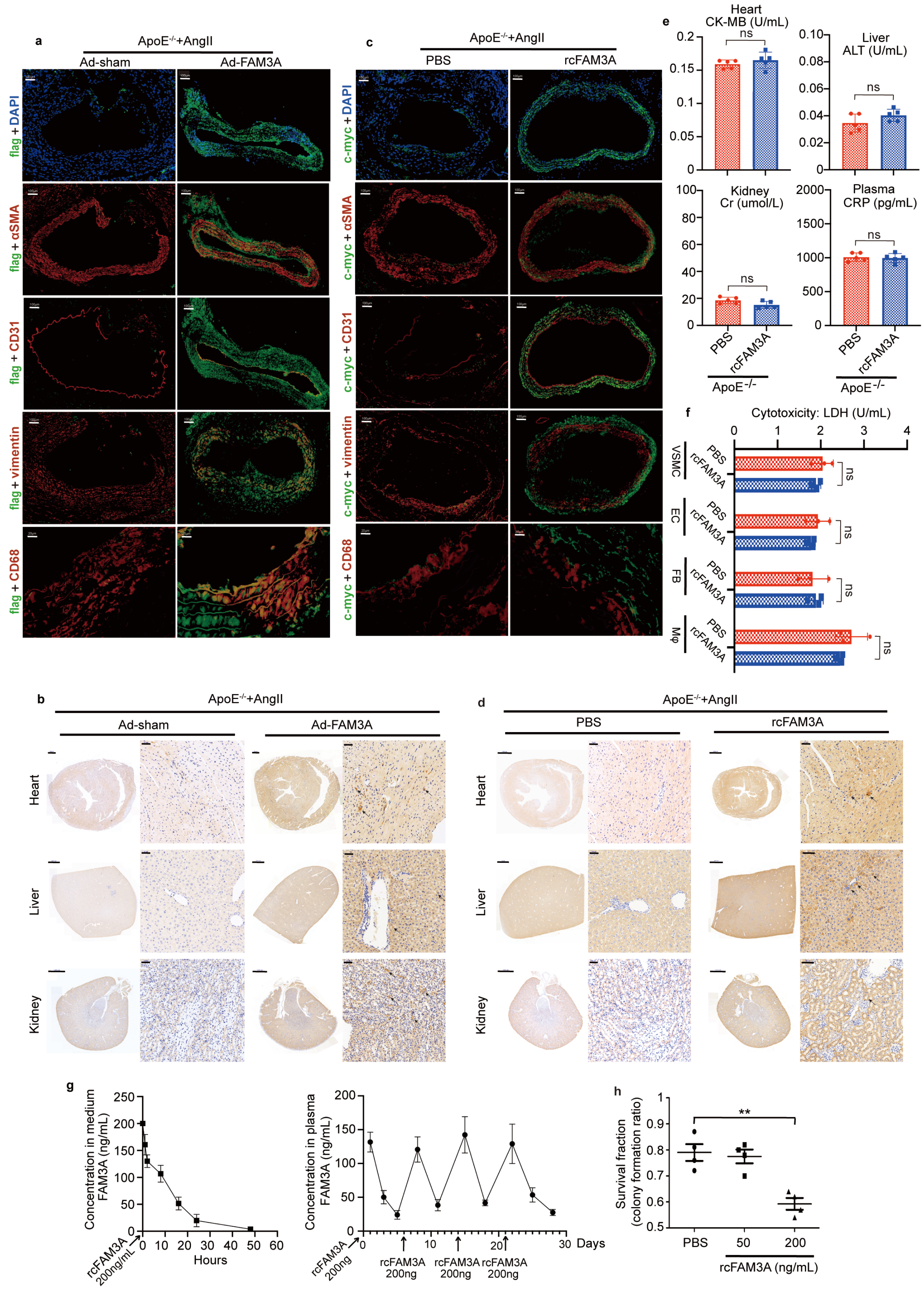

Supplementary Fig. 4 The influence of exogenous FAM3A in mice. a The cell-specific FAM3A location is shown by monitoring flag with immunefluorescence staining in aortas from AngII-ApoE$^{-/-}$ murine AAA models treated with or without FAM3A overexpression by adenovirus at 72-hour time point after tail vein injection (green indicates flag-FAM3A, red indicates αSMA: VSMCs, CD31: endothelial cells, vimentin: fibroblasts, or CD68: macrophages, respectively, and orange indicates flag^{+}αSMA^{+}, flag^{+}CD31^{+}, flag^{+}vimentin^{+}, or

flag^{+}CD68^{+}, respectively; Representative images of n=4 biologically independent animals). Scale bar: 100 μm (DAPI, αSMA, CD31, vimentin) and 20 μm (CD68). **b** The organ FAM3A location is shown by monitoring flag with histochemistry staining in mice treated as in a (brown indicates flag-FAM3A; Representative images of n=3 biologically independent animals). Scale bar: 1000 μm, insets: 50 μm. **c** The cell-specific FAM3A location is shown by monitoring c-myc with immunefluorescence staining in aortas from AngII-ApoE$^{-/-}$ murine AAA models treated with or without recombinant FAM3A at 48-hour time point after tail vein injection (green indicates c-myc-FAM3A, red indicates αSMA: VSMCs, CD31: endothelial cells, vimentin: fibroblasts, or CD68: macrophages, respectively, and orange indicates c-myc^{+}αSMA^{+}, c-myc^{+}CD31^{+}, c-myc^{+}vimentin^{+}, or c-myc^{+}CD68^{+}, respectively; Representative images of n=4 biologically independent animals). Scale bar: 100 μm (DAPI, αSMA, CD31, vimentin) and 20 μm (CD68). **d** The organ FAM3A location is shown by monitoring c-myc with histochemistry staining in mice treated as in c (brown indicates c-myc-FAM3A; Representative images of n=3 biologically independent animals). Scale bar: 1000 μm, insets: 50 μm. **e** The organ toxicity was determined by the indicated parameters in normal ApoE$^{-/-}$ mice treated with or without recombinant FAM3A (n=5 biologically independent animals). **f** The cytotoxicity was determined by LDH release in normal cultured primary human vascular smooth muscle cell (VSMC), endothelial cell (EC), fibroblast (FB), and macrophage (Mφ) treated with or without recombinant FAM3A for 48 hours (n=3 biologically independent experiments). **g** The VSMCs were cultured and treated with recombinant FAM3A, and then FAM3A levels in medium were determined by ELISAs at the indicated time points (left, n=3 biologically independent experiments). The plasma FAM3A levels in ApoE$^{-/-}$ mice treated with recombinant FAM3A were determined by ELISAs at the indicated time points (right, n=3 biologically independent animals). **h** The survival fraction of VSMCs treated with or without recombinant FAM3A was determined by colony formation assays (n=4 biologically independent experiments). Data are presented as mean±SEM (e, f, g, h). Statistical significance was calculated with two-tailed independent *t* test (e, f) and one-way ANOVA followed by Tukey post hoc test (h) and *P* values are indicated ($^{ns}P \geq 0.05$, $^{**}P \leq 0.01$). Source data are provided as a Source Data file.

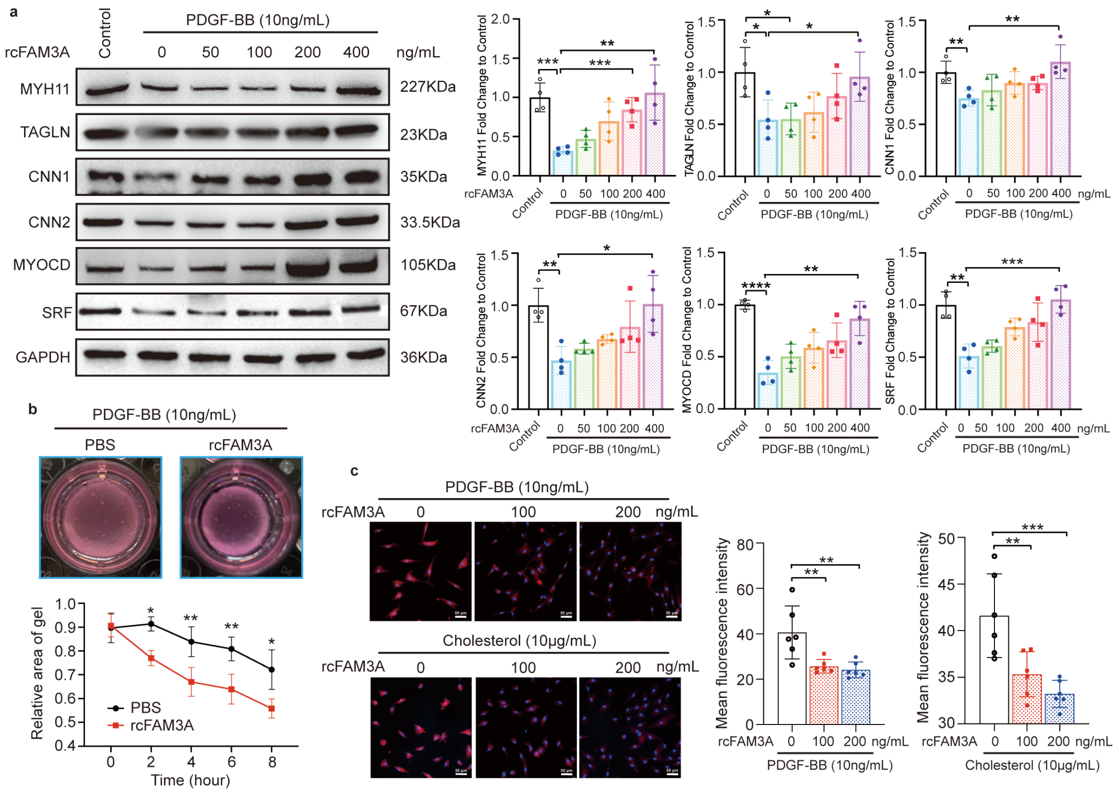

Supplementary Fig. 5 FAM3A maintains the contractile phenotype of VSMCs in vitro. VSMCs were treated with PDGF-BB (10 ng/mL) or cholesterol (10 ug/mL) for 48 hours, and meanwhile stimulated with recombinant FAM3A at indicated concentrations. **a** Representative western blot images and quantification of VSMC contractile marker proteins are shown (n=4 biologically independent experiments; quantitative comparisons between samples were run on the same gel). **b** VSMC contractility was quantified and shown (n=6 biologically independent experiments; **P*<0.05, ***P*<0.01 vs. parallel time control group). **c** Cellular ROS level was determined by MitoSOX™ Red mitochondrial superoxide indicator, and quantificatioin is shown (n=6 biologically independent experiments). Scale bar: 50 μm. Data are presented as mean±SEM (a-c). Statistical significance was calculated with a one-way ANOVA followed by Tukey post hoc test (a, c) and two-tailed independent *t* test (b) and *P* values are indicated (**P*<0. 05, ***P*≤0. 01, ****P*≤0. 001, *****P*≤0. 0001). Source data are provided as a Source Data file.

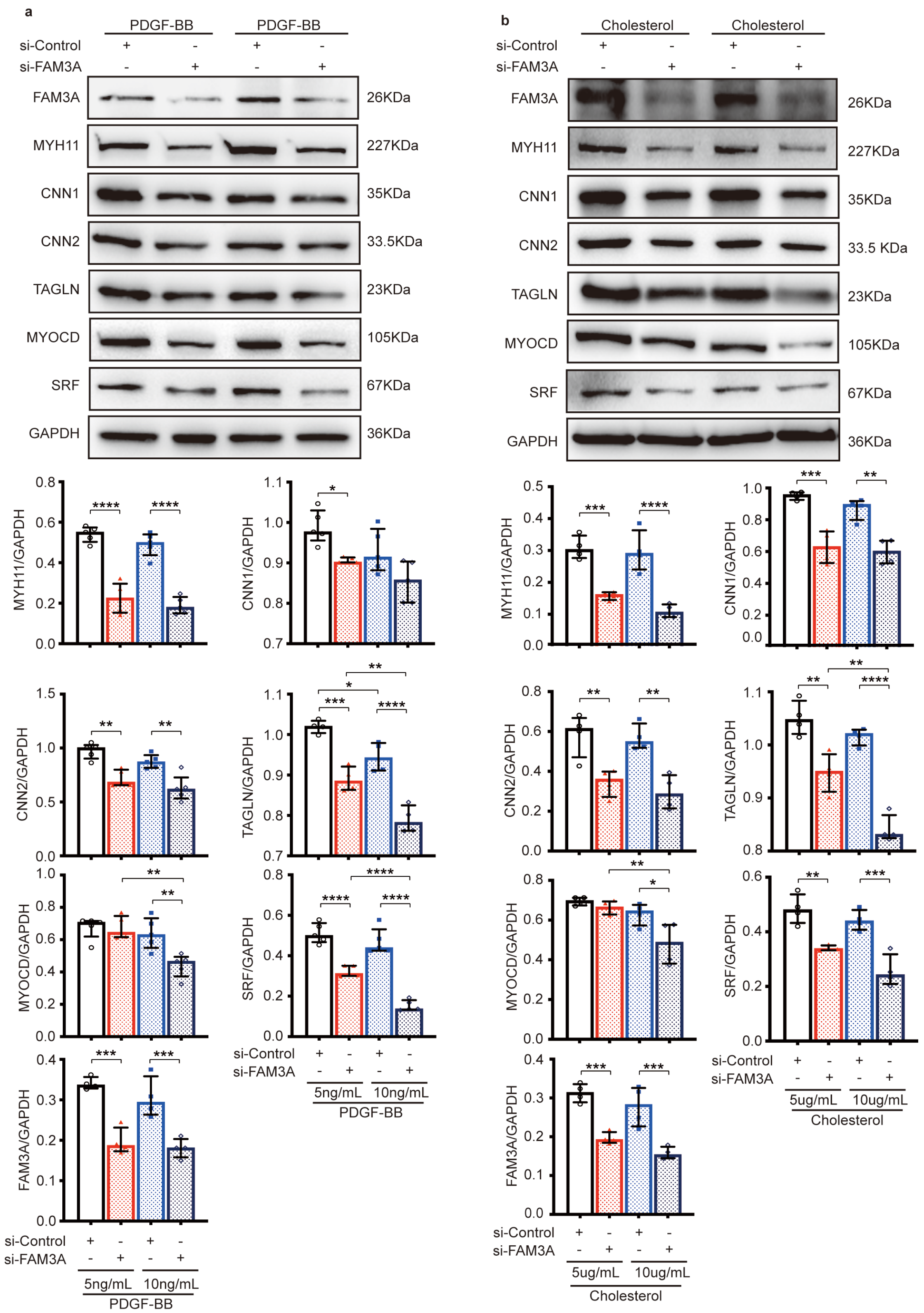

Supplementary Fig. 6 FAM3A ablation in VSMCs induces lower expression of contractile markers. a, b VSMCs were transfected with FAM3A siRNA or scramble control for 24 hours, and then treated with PDGF-BB (5 ng/mL or 10 ng/mL, a), or cholesterol (5 ug/mL or 10 ug/mL, b) for 48 hours. Representative western blot images and quantification of VSMC contractile marker proteins are shown (n=4 or 5 biologically independent experiments

for FAM3A or contractile marker proteins, respectively in a; n=4 biologically independent experiments in b; quantitative comparisons between samples were run on the same gel). Data are presented as mean±SEM (a, b). Statistical significance was calculated with one-way ANOVA followed by Tukey post hoc test (a, b) and *P* values are indicated (**P*<0. 05, ***P*≤0. 01, ****P*≤0. 001, *****P*≤0. 0001). Source data are provided as a Source Data file.

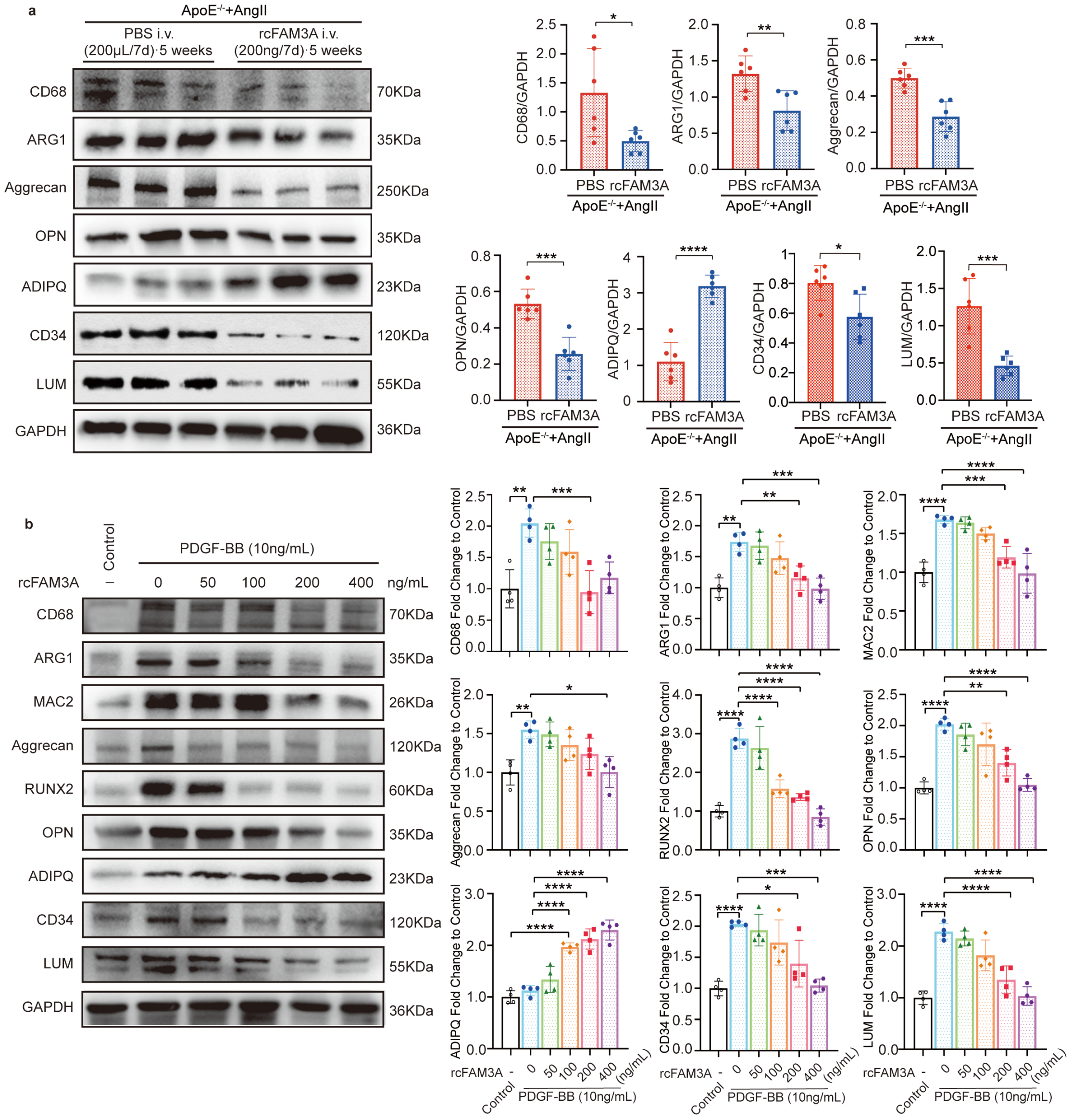

Supplementary Fig. 7 Recombinant FAM3A inhibits VSMC transdifferentiation toward intermediate cell types. a Representative western blot images and quantification of markers of macrophages (CD68 and ARG1), chondrocytes (Aggrecan), osteogenic cells (OPN), adipocytes (ADIPQ), mesenchymal cells (CD34), and fibroblasts (LUM) are shown in aortas from AngII-ApoE$^{-/-}$ murine AAA models supplemented with or without recombinant FAM3A (n=6 biologically independent animals). **b** VSMCs were treated with PDGF-BB (10 ng/mL) for 48 hours, and meanwhile stimulated with FAM3A at indicated concentrations. Representative western blot images and quantification of markers of macrophages (CD68, ARG1, and MAC2), chondrocytes (Aggrecan), osteogenic cells (RUNX2 and OPN), adipocytes (ADIPQ), mesenchymal cells (CD34), and fibroblasts (LUM) are shown (n=4 biologically independent experiments). Quantitative comparisons between samples were run on the same gel (a, b). Data are presented as mean±SEM (a, b). Statistical significance was calculated with two-tailed independent t test (a) and one-way ANOVA followed by Tukey post hoc test (b) and P values are indicated (*P<0. 05, **P≤0. 01, ***P≤0. 001, ****P≤0. 0001). Source data are provided as a Source Data file.

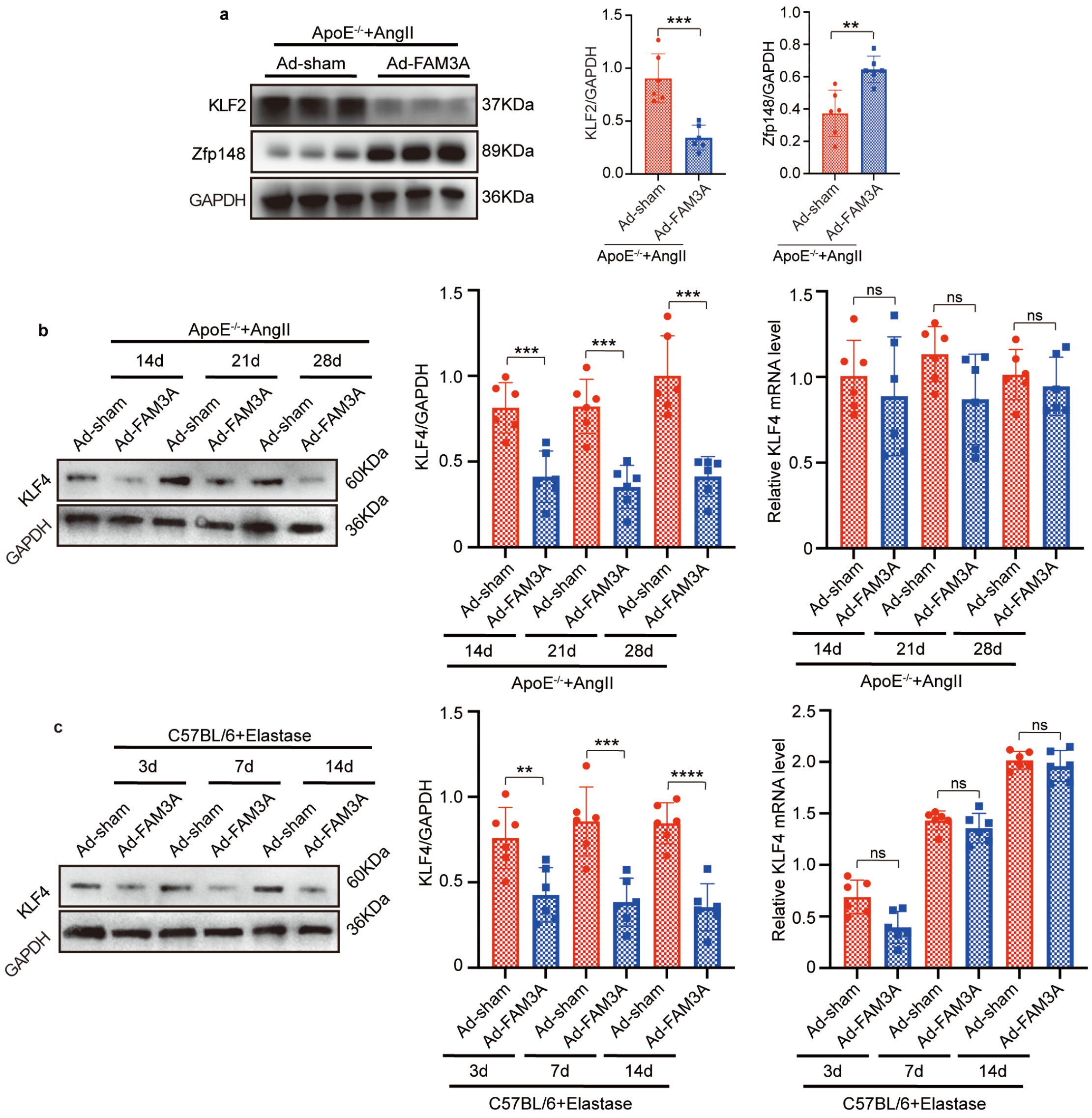

Supplementary Fig. 8 The KLF4 expression in murine AAA models. **a** Representative western blot images and quantification of KLF2 and Zfp148 in aortas from AngII-ApoE$^{-/-}$ murine AAA models treated with or without FAM3A overexpression by adenovirus (n=6 biologically independent animals). **b, c** Representative western blot images and quantification of KLF4 protein and mRNA levels in aortic specimens harvested at indicated time points from AngII-ApoE$^{-/-}$ and Elastase-C57BL/6 murine AAA models treated with or without FAM3A overexpression by adenovirus (n=6 biologically independent animals). Quantitative comparisons between samples were run on the same gel (a-c). Data are presented as mean±SEM (a-c). Statistical significance was calculated with two-tailed independent *t* test (a) and two-way ANOVA followed by Tukey post hoc test (b, c) and *P* values are indicated ($^{ns}P\geq0.05$, $^{**}P\leq0.01$, $^{***}P\leq0.001$, $^{****}P\leq0.0001$). Source data are provided as a Source Data file.

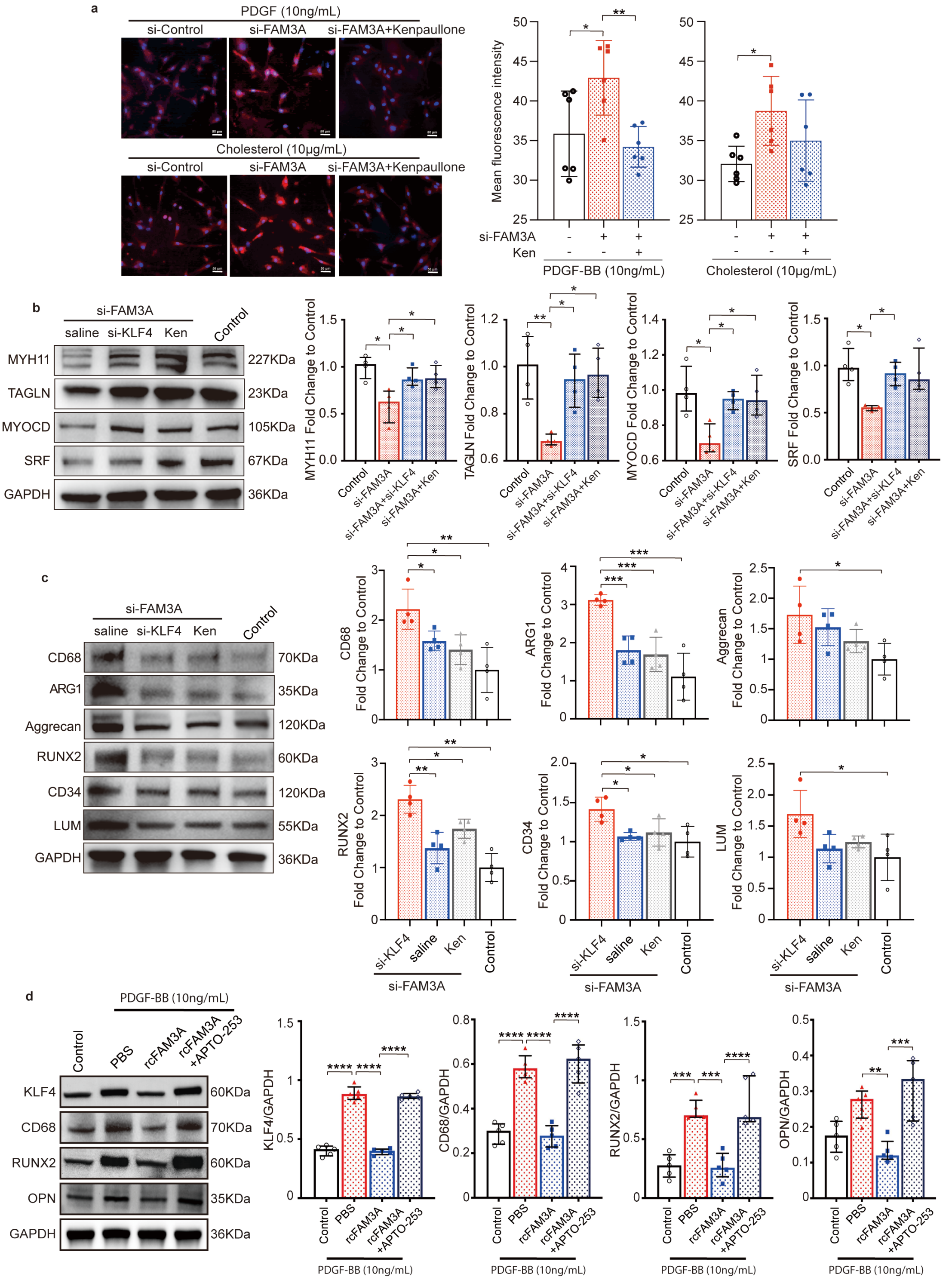

Supplementary Fig. 9 KLF4 signaling is involved in FAM3A biological function. a VSMCs were transfected with FAM3A siRNA and then treated with PDGF with or without KLF4 inhibitor (Kenpaullone). The cellular ROS level were detected and quantified (n=6 biologically independent experiments). Scale bar: 50 μm. **b, c** VSMCs were transfected with FAM3A siRNA and further transfected KLF4 siRNA or inhibitor (Ken). Representative western blot images and quantification of VSMC contractile and transdifferentiation markers are shown (n=4 biologically independent experiments). **d**, VSMCs were pretreated with KLF4 inducer APTO-253 (10 uM), and then stimulated with recombinant FAM3A (100 ng/mL) for

24 hours. Representative western blot images and quantification of KLF4, CD68, RUNX2, and OPN are shown (n=5 biologically independent experiments). Quantitative comparisons between samples were run on the same gel (b-d). Data are presented as mean±SEM (a-d). Statistical significance was calculated with one-way ANOVA followed by Tukey post hoc test (a-d) and *P* values are indicated (*$P<0.05$, **$P\leq 0.01$, ***$P\leq 0.001$, ****$P\leq 0.0001$). Source data are provided as a Source Data file.

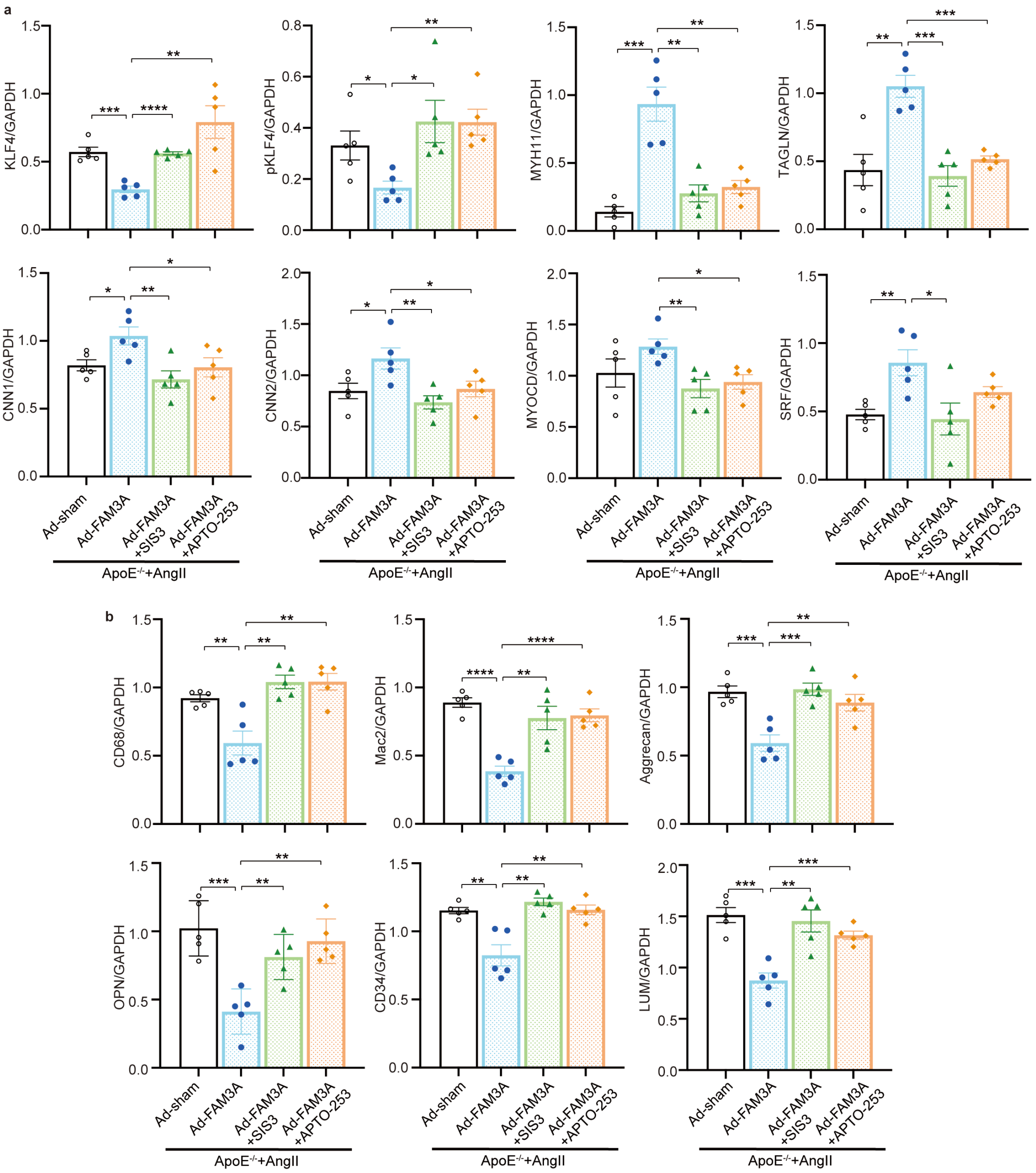

Supplementary Fig. 10 The TGFβ and KLF4 signaling is involved in the regulatory roles of FAM3A in VSMC phenotypes in vivo. a, b The quantification of the western blotting images in Fig. 8b (n=5 biologically independent animals, a) and 8c (n=5 biologically independent animals, b) is shown. Quantitative comparisons between samples were run on the same gel (a, b). Data are presented as mean±SEM (a, b). Statistical significance was calculated with one-way ANOVA followed by Tukey post hoc test (a, b) and *P* values are indicated (**P*<0. 05, ***P*≤0. 01, ****P*≤0. 001, *****P*≤0. 0001). Source data are provided as a Source Data file.

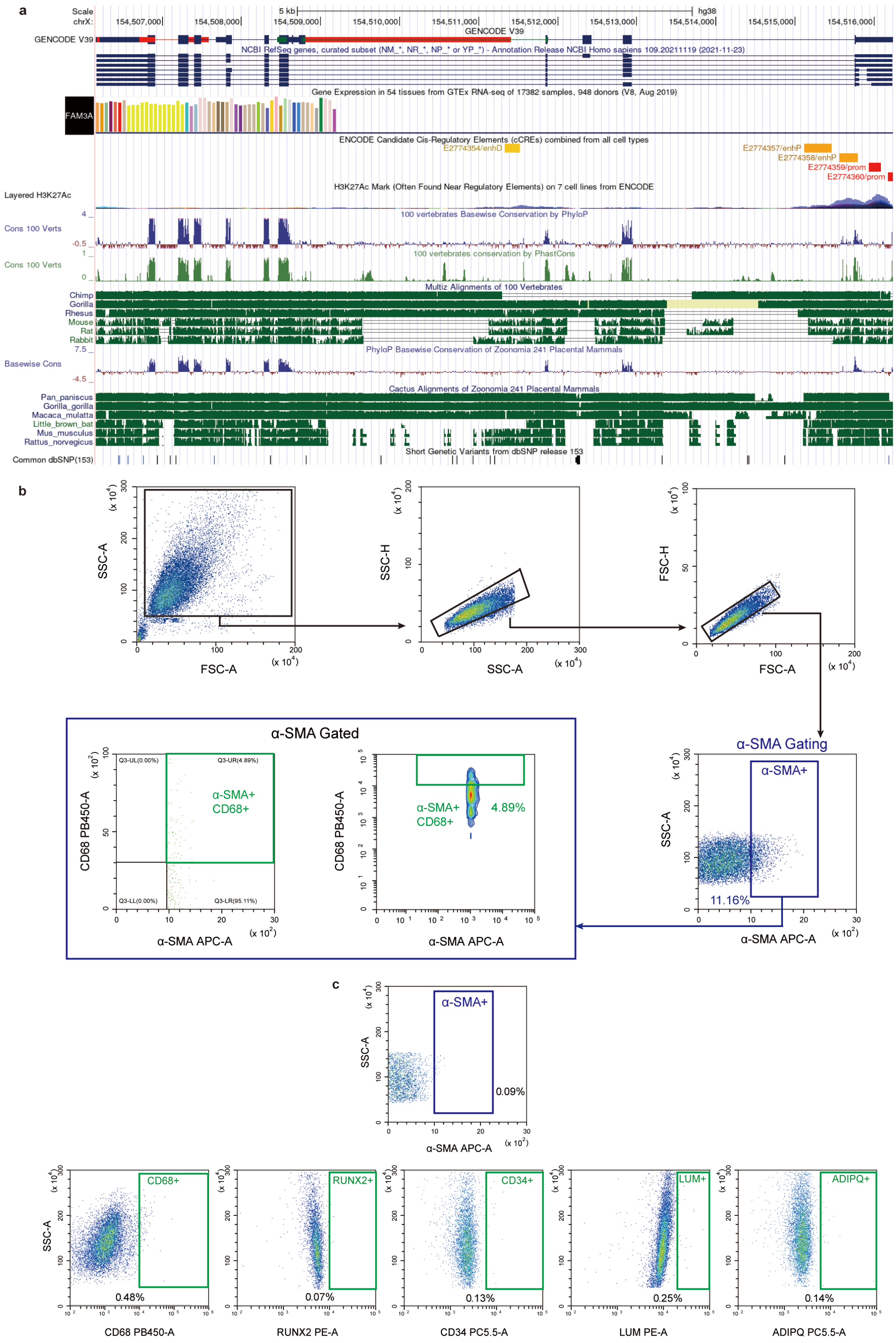

Supplementary Fig. 11 **a** Highly genetical conservation of FAM3A among mammalian species is shown (being computed at http://genome.ucsc.edu/). **b, c** Gating strategy to assess the percentage of transdifferentiated VSMCs. Flow cytometry of single cell suspension from saline or AngII treated ApoE$^{-/-}$ mouse aortas to screen out the transdifferentiated VSMCs.

Gating strategy includes: Forward vs Side Scatter to gate out debris and aggregates, α-SMA$^+$ gating, α-SMA$^+$ CD68$^+$ (representative experiment, n = 5). Other transdifferentiation markers (RUNX2, CD34, LUM, ADIPQ) were detected through the same gating strategy with slightly different gating determination threshold in the final gating. Sample load of single cell suspension ranged from 10,000 to 20,000 cells in each aorta. Cells were run on a CytoFLEX machine and analyzed with CytExpert (b). Fluorescence-minus-one (FMO) controls for transdifferentiated VSMC marker panel. All negative gates contain >99.5% of the cellular population (c).

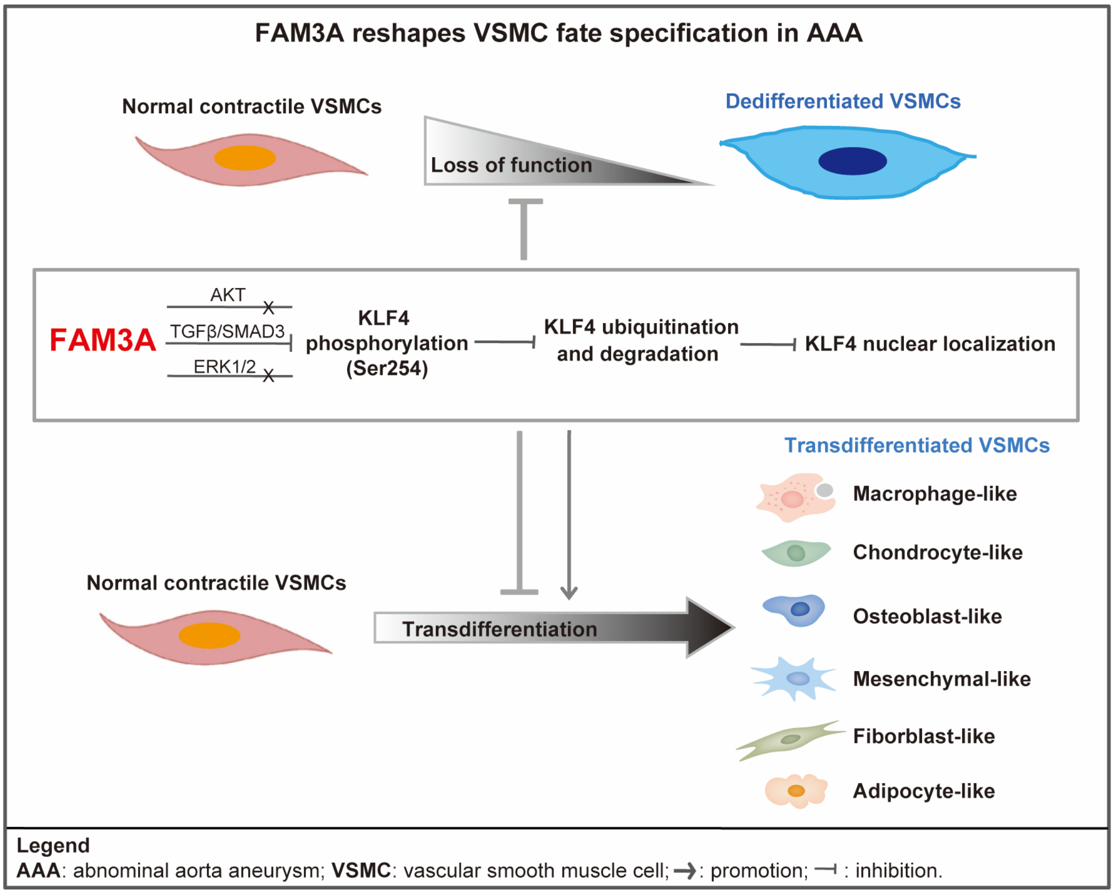

Supplementary Fig. 12 Schematic summary of FAM3A function in AAA. In the microenvironment of abdominal aortic aneurysm (AAA), FAM3A activates AKT, ERK1/2, and TGFβ/SMAD3 pathways. Through activation of TGFβ pathway, FAM3A suppresses KLF4 phosphorylation, elevates KLF4 ubiquitination and degradation, and decreases KLF4 protein expression and nuclear localization. Through suppressing KLF4 stability, FAM3A maintains well-differentiated status of VSMCs against loss-of-function phenotype (such as decreased MYH11, TAGLN, CNN1,2, SRF, MYOCD etc.), and protects VSMCs against transforming towards macrophage-like (markers such as CD68, ARG1, MAC2), chondrocyte-like (markers such as Aggrecan), osteoblast-like (markers such as OPN, RUNX2), mesenchymal-like (markers such as CD34), as well as fibroblast-like (markers such as LUM) cell subpopulations. However, FAM3A promotes an adipocyte-like (markers such as ADIPQ) transformation of VSMCs. Therefore, FAM3A is a regulator to reshape VSMC fate specification in the AAA microenvironment.

Supplementary Tables

Supplementary Table 1. Clinical characteristics of AAA patients and normal control

Terms	Control (n=6)	AAA patients (n=6)	*P* value[&]
Sex: Male, n (%)	5 (83.3)	5 (83.3)	1
Age, years (mean±SEM)	55.3±14.4	65.8±6.5	0.15
Hypertension, n (%)	1 (16.7)	3 (50.0)	0.55
Diabetes mellitus, n (%)	2 (33.3)	2 (33.3)	1
Hyperlipidemia, n (%)	0 (0)	5 (83.3)	0.015*
Smoking, n (%)	3 (50.0)	4 (66.7)	1
Alcohol, n (%)	1 (16.7)	3 (50.0)	0.55
CAD[#], n (%)	1 (16.7)	1 (16.7)	1
Stroke, n (%)	0 (0)	2 (33.3)	0.46
Atherosclerosis in aorta, n (%)	0 (0)	0 (0)	-
Atherosclerosis, n (%)	3 (50.0)	4 (66.7)	1

CAD: coronary artery disease; &: Statistical significance of Age was calculated with two-tailed independent *t* test and others were derived from Fisher's exact test, and *P* values were presented. * marked significant difference with $P<0.\ 05$.

Supplementary Table 2. Markers used to identify the type of VSMC differentiation.

**Markers**	**Cell type**
MYH11	Contractile vSMC[1-3]
CNN1	Contractile vSMC[1-3]
CNN2	Developingandcontractile vSMC[4,5]
TAGLN	Contractile vSMC[1-3]
MYOCD	Contractile vSMC (transcription factor)[6,7]
SRF	Contractile vSMC (transcription factor)[6,7]
Galectin 3/Mac 2	Macrophage-like vSMC[8,9]
CD68	Macrophage-like vSMC[10]
Arginase 1	Macrophage-like vSMC[11]
Aggrecan	Chondrogenic-like vSMC[12]
RUNX2	Osteogenic-like vSMC[13,14]
Osteopontin	Osteogenic-like vSMC[15,16]
Adiponectin	Adipocyte-like vSMC[17]
CD34	Mesenchymal-like vSMC[18,19]
LUM	Fibroblast-like vSMC[3,20]

Supplementary Table 3. PCR Primers for Quantitative RT-PCR

**Genes**	**Species**	**Forward Primer**	**Reverse Primer**
fam3a	M	CCAGCCCAGAGAACTCAGTG	GCAGCCCCGCTGACTATTC
fam3a	H	GTGTCACATGGATCGTGGTC	TGCTCAATCAGCATCTTGTCC
klf4	M	GGCGAGTCTGACATGGCTG	GCTGGACGCAGTGTCTTCTC
srf	M	CCTCCGTCCGCAATGGTTC	AACTCGGATTCGCTTCACTTC
myocd	M	AGGAAGTTCCGATCAGTCTTACA	GGTATTAAGCCTTGGTTAGCCAG
myh11	M	ATGAGGTGGTCGTGGAGTTG	GCCTGAGAAGTATCGCTCCC

cnn1	M	TCTGCACATTTTAACCGAGGTC	TCTGTTGCTGCCCATTTGAAG
tagln	M	CGGCGTCACCTCTATGATCCCA	GCCAGCTTGTTCTTTACTTCAGC

Major Resources Information

Supplementary Table 4. Experimental animals

**Species/Strain**	**Vendor or Source**	**Background Strain**	**Sex**
Wild type C57Bl/6N	VitalRiver	C57Bl/6N	Male
B6-ApoE tm1 Mice (ApoE)	BejingHFK Bioscience	C57Bl/6J	Male
B6-ApoE tm1 Mice (ApoE)	BejingHFK Bioscience	C57Bl/6J	Female

Supplementary Table 5. Cultured Cells and related reagents

**Cells or reagents**	**Vendor or Source**	**Catalog #**
Human aortic smooth muscle cells	Sciencell	6110
Human aortic endothelial cells	Sciencell	6100
Human aortic adventitia fibroblasts	Sciencell	6120
HEK293T cells	ATCC	crl-3216
Human peripheral blood macrophages	Procell Life Science&Technology Co., Ltd, China	CP-H264
SMCM	Sciencell	1101
SMCGS	Sciencell	1152
FBS	Sciencell	0010
ECM	Sciencell	1001
FM	Sciencell	2301
RPMI1640	Solarbio Science&Technology Co., Ltd, China	31800
P/S	Sciencell	0503
dmem/Ham F12	Sigma	D8473
DMEM	Solarbio Science&Technology Co., Ltd, China	12100

Supplementary Table 6. Antibodies

Target antigen	Vendor or Source	Catalog #
phospho-AKT (WB)	Cell Signaling Technology	9271S
AKT (WB)	Cell Signaling Technology	9272S
SRF (WB)	Abcam	ab252868
Mkl1/MRTFA (WB)	Novus	NBP1-88498
FAM3A (WB)	Origene	TA324017
MYH11 (WB)	Abcam	ab125884
MYOCD (WB)	R&D	MAB4028
KLF4 (WB)	Abcam	ab106629
TAGLN/Transgelin (WB)	Abcam	ab14106
CNN1 (WB)	Sigma	231R-16-RUO
CNN2 (WB)	Novus	NBP2-01325
Galectin 3/Mac 2 (WB)	Abcam	ab76245
Arginase 1 (WB)	BioLegend	678802
CD68 (WB)	Cell Signaling Technology	86985S
Aggrecan (WB)	Abcam	ab3778
Osteopontin (WB)	Abcam	ab63856
Adiponectin (WB)	Abcam	ab22554
LUM (WB)	Abcam	ab168348
CD34 (WB/IF)	Abcam	ab81289
Phospho-KLF4(Ser254) (WB)	Affinity	AF2437
Ubiquitin (WB)	Cell Signaling Technology	3936S
Smad3 (WB)	Abcam	ab40854
Phospho-Smad3(S423+S425) (WB)	Abcam	ab52903
GAPDH (WB)	Abcam	ab181602
MMP2 (WB)	Cell Signaling Technology	87809S
MMP9 (WB)	Abcam	ab283575
Phospho-p44/42 MAPK (Erk1/2) (Thr202/Tyr204) (WB)	Cell Signaling Technology	4370S
p44/42 MAPK (Erk1/2) (WB)	Cell Signaling Technology	4695S
RUNX2 (WB)	Cell Signaling Technology	12556S
Goat anti-rabbit IgG (HRP-conjugated, WB)	Cell Signaling Technology	7074
Horse anti-mouse IgG (HRP-conjugated, WB)	Cell Signaling Technology	7076
Flag (IHC/IF)	Abcam	ab205606
c-myc (IHC/IF)	Abcam	ab32072
KLF4 (IF)	Proteintech	11880-1-AP
SRF (IF)	Santa Cruz	sc-25290
αSMA-FITC conjugate (IF)	Invitrogen	F3777
αSMA (IF)	Abcam	ab7817
CD68 (IF)	Santa	sc-20060

RUNX2 (IF)	Abcam	ab192256
Vimentin (IF)	Proteintech	60330-1-Ig
LUM (IF)	Abcam	ab252925
CD31 (IF)	Santa	sc-376764
FAM3A (IF, human)	Sigma-Aldrich	HPA035268
FAM3A (IF, mouse)	Novus	NBP3-17844
ADIPQ (IF)	Proteintech	21613-1-AP
Aggrecan (IF)	Proteintech	13880-1-AP
HRP-conjugated Goat anti-rabbit (IHC)	Cell Signaling Technology	8114
HRP-conjugated Goat anti-mouse (IHC)	Cell Signaling Technology	8125
Alexa Fluor 488-conjugated Goat anti-Rabbit IgG (IF)	Invitrogen	A-11008
Alexa Fluor 594-conjugated Goat anti-Rabbit IgG (IF)	Invitrogen	A-11012
Alexa Fluor 488-conjugated Goat anti-Mouse IgG (IF)	Invitrogen	A-11029
Alexa Fluor 594-conjugated Goat anti-Mouse IgG (IF)	Invitrogen	A-11032
CoraLite Plus 647-conjugate α-SMA (FC)	Proteintech	CL647-67735
Brilliant Violet 421-anti-CD68 (FC)	BioLegend	137017
PerCP/Cyanine5.5-anti-CD34 (FC)	BioLegend	119328
LUM (FC)	Invitrogen	PA5-14570
ADIPQ (FC)	Abcam	ab181281
RUNX2-PE Conjugate (FC)	Cell Signaling Technology	98059S

WB: western blot; IF: immunofluorescence; IHC: immunohistochemistry; FC: flow cytometry.

Supplementary Table 7. Other important reagents

Name	Vendor or Source	Catalog #
Recombinant Human PDGF-BB Protein, CF	R&D	220-BB-050
Recombinant Human FAM3A	Origene	TP303495
Cholesterol, water soluble	MP Biomedicals	0219934230
FAM3A ELISA Kit (mouse)	lifespan	LS-F17398
FAM3A ELISA Kit (human)	lifespan	LS-F35367
Cytoplasma/nucleu extract Kit	Beyotime, China	P0027
Collagenase type I	Sigma	C0130
Collagenase type XI	Sigma	C7657
Hyaluronidase type I-S	Sigma	H3506
Deoxyribonuclease I	Sigma	D5025
Elastase type I	Sigma	E1250
R-PE Conjugation Kit	Abcam	ab102918

PerCP/Cy5.5 Conjugation Kit	Abcam	ab102911
DHE (Dihydroethidium) Assay Kit/Reactive Oxygen Species	Abcam	ab236206
MitoSOX™ Red mitochondrial superoxide indicator	Abmole	M19992
APTO-253	MCE	hy-16291
MK-2206 2HCl	MCE	hy-10358
U0126-EtOH	MCE	hy-12031
SIS3 HCl	MCE	hy-13013
Kenpaullone	MCE	HY12302
Cell contraction assay Kit	Cell Biolabs	CBA-201
IL1β ELISA Kit (mouse)	R&D	MLB00C
IL6 ELISA Kit (mouse)	R&D	M6000B
TNFα ELISA Kit (mouse)	R&D	MTA00B
Lipofectamine 2000	Invitrogen	11668-019
Oligofectamine	Invitrogen	12252-011
ViraPower Adenoviral Gateway Expression Kit	Invitrogen	K4930-00
Protein A/G magnetic beads	MCE	HY-K0202

Supplementary References

1 Owens, G. K., Kumar, M. S. & Wamhoff, B. R. Molecular regulation of vascular smooth muscle cell differentiation in development and disease. *Physiol Rev* **84**, 767-801, doi:10.1152/physrev.00041.2003 (2004).

2 Rzucidlo, E. M., Martin, K. A. & Powell, R. J. Regulation of vascular smooth muscle cell differentiation. *J Vasc Surg* **45 Suppl A**, A25-32, doi:10.1016/j.jvs.2007.03.001 (2007).

3 Wirka, R. C. *et al.* Atheroprotective roles of smooth muscle cell phenotypic modulation and the TCF21 disease gene as revealed by single-cell analysis. *Nat Med* **25**, 1280-1289, doi:10.1038/s41591-019-0512-5 (2019).

4 Hossain, M. M., Hwang, D. Y., Huang, Q. Q., Sasaki, Y. & Jin, J. P. Developmentally regulated expression of calponin isoforms and the effect of h2-calponin on cell proliferation. *Am J Physiol Cell Physiol* **284**, C156-167, doi:10.1152/ajpcell.00233.2002 (2003).

5 Tang, J. *et al.* A critical role for calponin 2 in vascular development. *J Biol Chem* **281**, 6664-6672, doi:10.1074/jbc.M506991200 (2006).

6 Long, X., Bell, R. D., Gerthoffer, W. T., Zlokovic, B. V. & Miano, J. M. Myocardin is sufficient for a smooth muscle-like contractile phenotype. *Arterioscler Thromb Vasc Biol* **28**, 1505-1510, doi:10.1161/ATVBAHA.108.166066 (2008).

7 Spin, J. M., Maegdefessel, L. & Tsao, P. S. Vascular smooth muscle cell phenotypic plasticity: focus on chromatin remodelling. *Cardiovasc Res* **95**, 147-155, doi:10.1093/cvr/cvs098 (2012).

8 Than, N. G. *et al.* A primate subfamily of galectins expressed at the maternal-fetal interface that promote immune cell death. *Proc Natl Acad Sci U S A* **106**, 9731-9736,

doi:10.1073/pnas.0903568106 (2009).
9 Zhang, M. *et al.* A Translocation Pathway for Vesicle-Mediated Unconventional Protein Secretion. *Cell* **181**, 637-652 e615, doi:10.1016/j.cell.2020.03.031 (2020).
10 Depuydt, M. A. C. *et al.* Microanatomy of the Human Atherosclerotic Plaque by Single-Cell Transcriptomics. *Circ Res* **127**, 1437-1455, doi:10.1161/CIRCRESAHA.120.316770 (2020).
11 Ohue-Kitano, R. *et al.* alpha-Linolenic acid-derived metabolites from gut lactic acid bacteria induce differentiation of anti-inflammatory M2 macrophages through G protein-coupled receptor 40. *FASEB J* **32**, 304-318, doi:10.1096/fj.201700273R (2018).
12 Schlichting, N. *et al.* Suitability of porcine chondrocyte micromass culture to model osteoarthritis in vitro. *Mol Pharm* **11**, 2092-2105, doi:10.1021/mp5000554 (2014).
13 Komori, T. Runx2, an inducer of osteoblast and chondrocyte differentiation. *Histochem Cell Biol* **149**, 313-323, doi:10.1007/s00418-018-1640-6 (2018).
14 Loebel, C. *et al.* In vitro osteogenic potential of human mesenchymal stem cells is predicted by Runx2/Sox9 ratio. *Tissue Eng Part A* **21**, 115-123, doi:10.1089/ten.TEA.2014.0096 (2015).
15 Durham, A. L., Speer, M. Y., Scatena, M., Giachelli, C. M. & Shanahan, C. M. Role of smooth muscle cells in vascular calcification: implications in atherosclerosis and arterial stiffness. *Cardiovasc Res* **114**, 590-600, doi:10.1093/cvr/cvy010 (2018).
16 Tyson, K. L. *et al.* Osteo/chondrocytic transcription factors and their target genes exhibit distinct patterns of expression in human arterial calcification. *Arterioscler Thromb Vasc Biol* **23**, 489-494, doi:10.1161/01.ATV.0000059406.92165.31 (2003).
17 Shamsi, F. *et al.* Vascular smooth muscle-derived Trpv1(+) progenitors are a source of cold-induced thermogenic adipocytes. *Nat Metab* **3**, 485-495, doi:10.1038/s42255-021-00373-z (2021).
18 Sidney, L. E., Branch, M. J., Dunphy, S. E., Dua, H. S. & Hopkinson, A. Concise review: evidence for CD34 as a common marker for diverse progenitors. *Stem Cells* **32**, 1380-1389, doi:10.1002/stem.1661 (2014).
19 Zhao, G. *et al.* Single-cell RNA sequencing reveals the cellular heterogeneity of aneurysmal infrarenal abdominal aorta. *Cardiovasc Res* **117**, 1402-1416, doi:10.1093/cvr/cvaa214 (2021).
20 Medley, S. C., Rathnakar, B. H., Georgescu, C., Wren, J. D. & Olson, L. E. Fibroblast-specific Stat1 deletion enhances the myofibroblast phenotype during tissue repair. *Wound Repair Regen* **28**, 448-459, doi:10.1111/wrr.12807 (2020).
