# Supplementary material for: FAM3A reshapes VSMC fate specification in abdominal aortic aneurysm by regulating KLF4 ubiquitination
Source: Nat Commun. 2023 Sep 2;14:5360. doi: 10.1038/s41467-023-41177-x (PMC10475135; doi:10.1038/s41467-023-41177-x)
Supplement: Supplementary file 3 — Reporting Summary [file 41467_2023_41177_MOESM3_ESM.pdf]

nature portfolio

Corresponding author(s): Dan Yang
Yuehong Zheng

Last updated by author(s): Aug 19, 2023

Reporting Summary

Nature Portfolio wishes to improve the reproducibility of the work that we publish. This form provides structure for consistency and transparency in reporting. For further information on Nature Portfolio policies, see our Editorial Policies and the Editorial Policy Checklist.

Statistics

For all statistical analyses, confirm that the following items are present in the figure legend, table legend, main text, or Methods section.

n/a	Confirmed	
☐	☒	The exact sample size (*n*) for each experimental group/condition, given as a discrete number and unit of measurement
☐	☒	A statement on whether measurements were taken from distinct samples or whether the same sample was measured repeatedly
☐	☒	The statistical test(s) used AND whether they are one- or two-sided<br>*Only common tests should be described solely by name; describe more complex techniques in the Methods section.*
☒	☐	A description of all covariates tested
☐	☒	A description of any assumptions or corrections, such as tests of normality and adjustment for multiple comparisons
☐	☒	A full description of the statistical parameters including central tendency (e.g. means) or other basic estimates (e.g. regression coefficient) AND variation (e.g. standard deviation) or associated estimates of uncertainty (e.g. confidence intervals)
☐	☒	For null hypothesis testing, the test statistic (e.g. *F*, *t*, *r*) with confidence intervals, effect sizes, degrees of freedom and *P* value noted<br>*Give P values as exact values whenever suitable.*
☒	☐	For Bayesian analysis, information on the choice of priors and Markov chain Monte Carlo settings
☒	☐	For hierarchical and complex designs, identification of the appropriate level for tests and full reporting of outcomes
☐	☒	Estimates of effect sizes (e.g. Cohen's *d*, Pearson's *r*), indicating how they were calculated

Our web collection on statistics for biologists contains articles on many of the points above.

Software and code

Policy information about availability of computer code

Data collection	Panoramic scanning system (Pannoramic MIDI, 3D HISTECH) was used to acquire immunofluorescence image.<br>SuperSignal™ West Pico PLUS Chemiluminescent System was used to acquire western blot images.<br>CFX384 Real-Time PCR Detection Systems were used to acquire qRT-PCR results.<br>All-in-one microplate reader software Gen5 2.07 was used to acquire ELISA assay results.<br>CytoFLEX (BeckmanCoulter, Inc, Bria, CA) was used to acquire Flow cytometry results.
Data analysis	-Immunofluorescence images were contrasted, overlaid, and analyzed with NIS ElementsAR ver. 4.6.0.<br>-Western blot images were contrasted and analyzed with Image Lab (Bio-Rad) and ImageJ ver. 1.53.<br>-Real-Time PCR results were analyzed with CFX manager ver. 3.1 (Bio-Rad).<br>-ELISA results were analyzed with Gen5 2.07.<br>-Flow cytometry results were analyzed with CytExpert ver. 2.4.0.28 (Beckman Coulter, Inc, Bria, CA).

For manuscripts utilizing custom algorithms or software that are central to the research but not yet described in published literature, software must be made available to editors and reviewers. We strongly encourage code deposition in a community repository (e.g. GitHub). See the Nature Portfolio guidelines for submitting code & software for further information.

Data

Policy information about availability of data

All manuscripts must include a data availability statement. This statement should provide the following information, where applicable:

- Accession codes, unique identifiers, or web links for publicly available datasets
- A description of any restrictions on data availability
- For clinical datasets or third party data, please ensure that the statement adheres to our policy

All data supporting the findings of this study are available in the Source Data file. The uncropped western blots are shown in the Supplementary Information. FASTQ files from the RNA-seq performed on freshly isolated aortas in AngII-ApoE-/- mice with AAA described in this paper have been deposited in the Gene Expression Omnibus (GEO) database under the accession code GSE230163 [https://www.ncbi.nlm.nih.gov/geo/query/acc.cgi?acc=GSE230163]. The GRCm38 data in this study is available at the NIH GenBank repository website [https://www.ncbi.nlm.nih.gov/datasets/genome/GCF_000001635.20/]. Public transcript microarray datasets from AAA patients and normal control subjects were downloaded from the GEO database under the accession codes GSE47472 [https://www.ncbi.nlm.nih.gov/geo/query/acc.cgi?acc=GSE47472] and GSE57691 [https://www.ncbi.nlm.nih.gov/geo/query/acc.cgi?acc=GSE57691]. Public single-cell RNA transcript seq data from AngII-ApoE-/- mice with AAA and saline-ApoE-/- control mice was downloaded from the Genome Sequence Archive (GSA) under the code PRJCA006049 [https://ngdc.cncb.ac.cn/gsa/PRJCA006049]. Public single-cell RNA transcript seq data from Cacl2-C57BL/6 mice with AAA and saline-C57BL/6 control mice was downloaded from the GEO under the code GSE164678 [https://www.ncbi.nlm.nih.gov/geo/query/acc.cgi?acc=GSE164678]. Source data are provided with this paper.

Research involving human participants, their data, or biological material

Policy information about studies with human participants or human data. See also policy information about sex, gender (identity/presentation), and sexual orientation and race, ethnicity and racism.

Reporting on sex and gender	In our study, FAM3A expression levels were detected in 6 AAA patients and 6 normal individuals. The sex/gender was considered in our study design. Additionaly, epidemiologically speaking, the incidence of AAA disease is higher among male than female people. So the specimens collected from male AAA patients was more than female.
Reporting on race, ethnicity, or other socially relevant groupings	None
Population characteristics	The mean age of disease group was 65.8±6.5y with the dilated aortic diameter over 5.5 cm. The mean age of control group was 55.3±14.4y. The information for Gender, Hypertension, Diabetes mellitus, Smoking, Coronary artery disease, and Atherosclerosis was not statistically significant between disease and control groups. Detailed clinical characteristics are summarized in Supplementary Table 1.
Recruitment	Patients admitted in our center that underwent open abdominal aortic aneurysm resection (the dilated aorta diameter > 5.5 cm) were recruited in this study without selection bias, and their aneurysm tissues were obtained. The control aorta tissues were obtained from the normal aorta of individuals who underwent in situ autologous renal transplantation for renal artery stenosis. Based on the disease characteristics of abdominal aortic aneurysm per se, old age and male are high risk factors. Therefore, we matched the items of sex and age between these two groups by propensity score in a ratio of 1:1, and six individuals each were eventually enrolled in this study.
Ethics oversight	The use of human specimens was approved by the Ethics Review Board of Peking Union Medical College Hospital, Beijing, China. Written informed consent was obtained from all patients included in the study.

Note that full information on the approval of the study protocol must also be provided in the manuscript.

Field-specific reporting

Please select the one below that is the best fit for your research. If you are not sure, read the appropriate sections before making your selection.

☒ Life sciences ☐ Behavioural & social sciences ☐ Ecological, evolutionary & environmental sciences

For a reference copy of the document with all sections, see nature.com/documents/nr-reporting-summary-flat.pdf

Life sciences study design

All studies must disclose on these points even when the disclosure is negative.

Sample size	In general, no calculations were done to determine sample size. Sample size was chosen based on literature and variability observed in previous experience in the laboratory. In this study, sample size was determined based on the standards for cell experiments attempting to have a minimum of N = 3 biological independent samples and animal experiments attempting to have a minimum of N = 5 biological independent samples with sufficient reproducibility. Quantitative analysis of confocal images included replicates of different field scans per independent sample. The determination of sample size chosen in our study did not include the high-throughput experiments with big dataset from public database. The details on the sample size were included in each figure legend.
Data exclusions	Data were not excluded from analysis.

Replication	In this study, each result described in the paper is based on at least three independent biologicalreplicates but very often an experiment is based on more than three experiments. Figure legends indicate the number of independent experiments performed in each analysis.
Randomization	For all the experiments, samples were randomly allocated to experimental and control groups.
Blinding	For all the animal and cell experiments, the investigators were blinded to the group allocations during the measurements and data analysis, and the samples were tested in a randomized order. The blinding was not relevant in obtaining the aorta tissues and population characteristics from control and aneurysm individuals (those measurements and data analysis complying with blinding), as investigators should consider clinical characteristics.

Reporting for specific materials, systems and methods

We require information from authors about some types of materials, experimental systems and methods used in many studies. Here, indicate whether each material, system or method listed is relevant to your study. If you are not sure if a list item applies to your research, read the appropriate section before selecting a response.

Materials & experimental systems

n/a	Involved in the study
☐	☒ Antibodies
☐	☒ Eukaryotic cell lines
☒	☐ Palaeontology and archaeology
☐	☒ Animals and other organisms
☒	☐ Clinical data
☒	☐ Dual use research of concern
☒	☐ Plants

Methods

n/a	Involved in the study
☒	☐ ChIP-seq
☐	☒ Flow cytometry
☒	☐ MRI-based neuroimaging

Antibodies

Antibodies used

1. Rabbit polyclonal anti-phospho-AKT, Cell Signaling Technology, Cat#9271S; dilution 1:1000 for western blotting.
2. Rabbit polyclonal anti-AKT, Cell Signaling Technology, Cat#9272S; dilution 1:1000 for western blotting.
3. Rat monoclonal anti-SRF, Abcam, Cat#ab252868; dilution 1:1000 for western blotting.
4. Rabbit polyclonal anti-Mkl1/MRTFA, Novus, Cat#NBP1-88498; dilution 1:1000 for western blotting.
5. Rabbit polyclonal anti-FAM3A, Sigma-Aldrich, Cat#HPA035268; dilution 1:1000 for immunofluorescence (human tissue).
6. Rabbit polyclonal anti-MYH11, Abcam, Cat#ab125884; dilution 1:1000 for western blotting.
7. Mouse monoclonal anti-MYOCD, R & D, Cat#MAB4028; dilution 1:1000 for western blotting.
8. Rabbit polyclonal anti-KLF4, Abcam, Cat#ab106629; dilution 1:1000 for western blotting.
9. Rabbit polyclonal anti-TAGLN/Transgelin, Abcam, Cat#ab14106; dilution 1:1000 for western blotting.
10. Rabbit monoclonal anti-CNN1/Calponin-1, Sigma, Cat#231R-16-RUO; dilution 1:200 for western blotting.
11. Mouse monoclonal anti-CNN2/ Calponin-2, Novus, Cat#NBP2-01325; dilution 1:1000 for western blotting.
12. Rabbit monoclonal anti-Galectin 3/Mac 2, Abcam, Cat#ab76245; dilution 1:1000 for western blotting.
13. Mouse monoclonal anti-ARG1/Arginase 1, BioLegend, Cat#678802; dilution 1:1000 for western blotting.
14. Rabbit monoclonal anti-CD68, Cell Signaling Technology, Cat#86985S; dilution 1:1000 for western blotting.
15. Mouse monoclonal anti-Aggrecan, Abcam, Cat#ab3778; dilution 1:1000 for western blotting.
16. Rabbit polyclonal anti-OPN/Osteoponin, Abcam, Cat#ab63856; dilution 1:1000 for western blotting.
17. Mouse monoclonal anti-ADIPQ/Adiponectin, Abcam, Cat#ab22554; dilution 1:1000 for western blotting.
18. Rabbit monoclonal anti-LUM/Lumican, Abcam, Cat#ab168348; dilution 1:1000 for western blotting.
19. Rabbit monoclonal anti-CD34, Abcam, Cat#ab81289; dilution 1:5000 for western blotting, and dilution 1:1000 for immunofluorescence.
20. Goat polyclonal anti-phospho-KLF4(Ser254), Affinity, Cat#AF2437; dilution 1:1000 for western blotting.
21. Mouse monoclonal anti-Ubiquitin, Cell Signaling Technology, Cat#3936S; dilution 1:1000 for western blotting.
22. Rabbit monoclonal anti-Smad3, Abcam, Cat#ab40854; dilution 1:1000 for western blotting.
23. Rabbit monoclonal anti-phospho-Smad3(S423+S425), Abcam, Cat#ab52903; dilution 1:1000 for western blotting.
24. Rabbit monoclonal anti-GAPDH, Abcam, Cat#ab181602; dilution 1:1000 for western blotting.
25. Rabbit monoclonal anti-MMP2, Cell Signaling Technology, Cat#87809S; dilution 1:1000 for western blotting.
26. Rabbit recombinant multiclonal anti-MMP9, Abcam, Cat#ab283575; dilution 1:1000 for western blotting.
27. Rabbit monoclonal anti-Phospho-p44/42 MAPK (Erk1/2) (Thr202/Tyr204), Cell Signaling Technology, Cat#4370S; dilution 1:1000 for western blotting.
28. Rabbit monoclonal anti-p44/42 MAPK (Erk1/2), Cell Signaling Technology, Cat#4695S; dilution 1:1000 for western blotting.
29. Rabbit monoclonal anti-RUNX2, Cell Signaling Technology, Cat#12556S; dilution 1:1000 for western blotting.
30. Rabbit polyclonal anti-KLF4, Proteintech, Cat#11880-1-AP; dilution 1:100 for immunofluorescence.
31. Rabbit monoclonal anti-Flag, Abcam, Cat#ab205606; dilution 1:500 for immunohistochemistry and immunofluorescence.
32. Mouse monoclonal anti-SRF, Santa, Cat#sc-25290; dilution 1:100 for immunofluorescence.
33. Rabbit monoclonal anti-c-myc, Abcam, Cat#ab32072; dilution 1:100 for immunohistochemistry and immunofluorescence.
34. Mouse monoclonal anti-α-SMA, Abcam, Cat#ab7817; dilution 1:100 for immunofluorescence.
35. Mouse monoclonal anti-CD68, Santa, Cat#sc-20060; dilution 1:100 for immunofluorescence.
36. Rabbit monoclonal anti-RUNX2, Abcam, Cat#ab192256; dilution 1:1000 for immunofluorescence.
37. Rabbit monoclonal anti-LUM/Lumican, Abcam, Cat#ab252925; dilution 1:4000 for immunofluorescence.
38. Rabbit polyclonal anti-LUM/Lumican, Invitrogen, Cat#PA5-14570; dilution 1:50 for flow cytometry.

39. Mouse monoclonal anti-Vimentin, Proteintech, Cat#60330-1-Ig; dilution 1:5000 for immunofluorescence.
40. Mouse monoclonal anti-CD31/PECAM-1, Santa, Cat#sc-376764; dilution 1:100 for immunofluorescence.
41. Rabbit polyclonal anti-ADIPQ/Adiponectin, Proteintech, Cat#21613-1-AP; dilution 1:100 for immunofluorescence.
42. Rabbit polyclonal anti-Aggrecan, Proteintech, Cat#13880-1-AP; dilution 1:500 for immunofluorescence.
43. Rat monoclonal Brilliant Violet 421-anti-mouse CD68, BioLegend, Cat#137017; dilution 1:100 for flow cytometry.
44. Rabbit monoclonal RUNX2(D1L7F) (PE Conjugate), Cell Signaling Technology, Cat#98059S; dilution 1:50 for flow cytometry.
45. Rat monoclonal PerCP/Cyanine5.5-anti-mouse CD34, BioLegend, Cat#119328; dilution 1:100 for flow cytometry.
46. Mouse monoclonal CoraLite Plus 647-conjugated anti-α-SMA, Peoteintech, Cat#CL647-67735; dilution 1:100 for flow cytometry.
47. Rabbit monoclonal anti-ADIPQ/Adiponectin, Abcam, Cat#ab181281; dilution 1:50 for flow cytometry.
48. Mouse monoclonal FITC-conjugated anti-α-SMA, Sigma-Aldrich, Cat#F3777; dilution 1:100 for immunofluorescence.
49. Rabbit polyclonal anti-FAM3A, Origene, Cat#TA324017; dilution 1:1000 for western blotting.
50. Rabbit polyclonal anti-FAM3A, Novus, Cat#NBP3-17844; dilution 1:100 for immunofluorescence.
51. HRP-linked Goat anti-Rabbit IgG, Cell Signaling Technology, Cat#7074; dilution 1:3000 for western blotting.
52. HRP-linked Goat anti-Mouse IgG, Cell Signaling Technology, Cat#7076; dilution 1:3000 for western blotting.
53. HRP-linked Goat anti-Rabbit IgG, Cell Signaling Technology, Cat#8114; being diluted by the manufacturer for immunohistochemistry.
54. HRP-linked Goat anti-Mouse IgG, Cell Signaling Technology, Cat#8125; being diluted by the manufacturer for immunohistochemistry.
55. Alexa Fluor 488-conjugated Goat anti-Rabbit IgG, Invitrogen, Cat#A-11008; dilution 1:500 for immunofluorescence.
56. Alexa Fluor 594-conjugated Goat anti-Rabbit IgG: Invitrogen, Cat#A-11012; dilution 1:1000 for immunofluorescence.
57. Alexa Fluor 488-conjugated Goat anti-Mouse IgG: Invitrogen, Cat#A-11029; dilution 1:200 for immunofluorescence.
58. Alexa Fluor 594-conjugated Goat anti-Mouse IgG: Invitrogen, Cat#A-11029, dilution 1:1000 for immunofluorescence.

Validation

1. https://www.cellsignal.com/products/primary-antibodies/phospho-akt-ser473-antibody/9271
2. https://www.cellsignal.com/products/primary-antibodies/akt-antibody/9272
3. https://www.abcam.cn/products/primary-antibodies/serum-response-factor-srf-antibody-2c5-ab252868.html
4. https://www.novusbio.com/products/mkl1-antibody_nbp1-88498
5. https://www.sigmaaldrich.cn/CN/zh/product/sigma/hpa035268
6. https://www.abcam.cn/products/primary-antibodies/smooth-muscle-myosin-heavy-chain-11-antibody-ab125884.html
7. https://www.rndsystems.com/cn/products/human-mouse-myocardin-antibody-355521_mab4028
8. https://www.abcam.cn/products/primary-antibodies/klf4-antibody-ab106629.html
9. https://www.abcam.cn/products/primary-antibodies/taglntransgelin-antibody-ab14106.html
10. https://www.cellmarque.com/antibodies/CM/1975/Calponin-1_EP798Y
11. https://www.novusbio.com/products/calponin-2-antibody-oti2b5_nbp2-01325
12. https://www.abcam.cn/products/primary-antibodies/galectin-3-antibody-ep2775y-ab76245.html
13. https://www.biolegend.com/en-us/products/purified-anti-arginase-1-antibody-12106
14. https://www.cellsignal.com/products/primary-antibodies/cd68-multimab-rabbit-mab-mix/86985
15. https://www.abcam.cn/products/primary-antibodies/aggrecan-antibody-6-b-4-ab3778.html
16. https://www.abcam.cn/products/primary-antibodies/osteopontin-antibody-ab63856.html
17. https://www.abcam.cn/products/primary-antibodies/adiponectin-antibody-19f1-ab22554.html
18. https://www.abcam.cn/products/primary-antibodies/lumican-antibody-epr88982-ab168348.html
19. https://www.abcam.cn/products/primary-antibodies/cd34-antibody-ep373y-ab81289.html
20. https://www.rndsystems.com/cn/products/mouse-dr3-tnfrsf25-antibody_af2437
21. https://www.cellsignal.com/products/primary-antibodies/ubiquitin-p4d1-mouse-mab/3936
22. https://www.abcam.cn/products/primary-antibodies/smad3-antibody-ep568y-ab40854.html
23. https://www.abcam.cn/products/primary-antibodies/smad3-phospho-s423--s425-antibody-ep823y-ab52903.html
24. https://www.abcam.cn/products/primary-antibodies/gapdh-antibody-epr16891-loading-control-ab181602.html
25. https://www.cellsignal.com/products/primary-antibodies/mmp-2-d2o4t-rabbit-mab/87809
26. https://www.abcam.cn/products/primary-antibodies/mmp9-antibody-rm1020-ab283575.html
27. https://www.cellsignal.com/products/primary-antibodies/phospho-p44-42-mapk-erk1-2-thr202-tyr204-d13-14-4e-xp-rabbit-mab/4370
28. https://www.cellsignal.com/products/primary-antibodies/p44-42-mapk-erk1-2-137f5-rabbit-mab/4695
29. https://www.cellsignal.com/products/primary-antibodies/runx2-d1l7f-rabbit-mab/12556
30. https://www.ptgcn.com/Products/KLF4-Antibody-11880-1-AP.htm
31. https://www.abcam.cn/products/primary-antibodies/ddddk-tag-binds-to-flag-tag-sequence-antibody-epr20018-251-ab205606.html
32. https://www.scbt.com/p/srf-antibody-a-11/
33. https://www.abcam.cn/products/primary-antibodies/c-myc-antibody-y69-chip-grade-ab32072.html
34. https://www.abcam.cn/products/primary-antibodies/alpha-smooth-muscle-actin-antibody-1a4-ab7817.html
35. https://datasheets.scbt.com/sc-20060.pdf
36. https://www.abcam.cn/products/primary-antibodies/runx2-antibody-epr14334-ab192256.html
37. https://www.abcam.cn/products/primary-antibodies/lumican-antibody-epr22511-63-ab252925.html
38. https://www.thermofisher.cn/cn/zh/antibody/product/LUM-Antibody-Polyclonal/PA5-14570
39. https://www.ptgcn.com/products/pictures/pdf/60330-1-Ig.pdf
40. https://datasheets.scbt.com/sc-376764.pdf
41. https://www.thermofisher.cn/cn/zh/antibody/product/ADIPOQ-Antibody-Polyclonal/21613-1-AP
42. https://www.ptgcn.com/products/pictures/pdf/13880-1-AP.pdf
43. https://www.biolegend.com/en-us/products/brilliant-violet-421-anti-mouse-cd68-antibody-9125
44. https://www.cellsignal.cn/products/antibody-conjugates/runx2-d1l7f-rabbit-mab-pe-conjugate/98059
45. https://www.biolegend.com/en-us/products/percp-cyanine5-5-anti-mouse-cd34-antibody-15659
46. https://www.ptgcn.com/products/pictures/pdf/CL647-67735.pdf
47. https://www.abcam.cn/products/primary-antibodies/adiponectin-antibody-epr17019-ab181281.html
48. https://www.sigmaaldrich.cn/CN/zh/product/sigma/f3777
49. https://www.origene.com.cn/catalog/antibodies/primary-antibodies/ta324017/fam3a-rabbit-polyclonal-antibody
50. https://www.novusbio.com/products/fam3a-antibody_nbp3-17844
51. https://www.cellsignal.com/products/secondary-antibodies/anti-rabbit-igg-hrp-linked-antibody/7074

52. https://www.cellsignal.com/products/secondary-antibodies/anti-rabbit-igg-hrp-linked-antibody/7076
53. https://www.cellsignal.com/products/secondary-antibodies/boost-ihc-detection-reagent-hrp-rabbit/8114
54. https://www.cellsignal.com/products/secondary-antibodies/boost-ihc-detection-reagent-hrp-rabbit/8125
55. https://www.thermofisher.cn/cn/zh/antibody/product/Goat-anti-Rabbit-IgG-H-L-Cross-Adsorbed-Secondary-Antibody-Polyclonal/A-11008
56. https://www.thermofisher.cn/cn/zh/antibody/product/Goat-anti-Rabbit-IgG-H-L-Cross-Adsorbed-Secondary-Antibody-Polyclonal/A-11012
57. https://www.thermofisher.cn/cn/zh/antibody/product/Goat-anti-Mouse-IgG-H-L-Highly-Cross-Adsorbed-Secondary-Antibody-Polyclonal/A-11029
58. https://www.thermofisher.cn/cn/zh/antibody/product/Goat-anti-Mouse-IgG-H-L-Highly-Cross-Adsorbed-Secondary-Antibody-Polyclonal/A-11032

Eukaryotic cell lines

Policy information about cell lines and Sex and Gender in Research

Cell line source(s)	aortic SMCs (human, Sciencell Cat#6110); aortic ECs (human, Sciencell Cat#6100); fibroblasts (human, Sciencell Cat#6120); HK293T (human, ATCC Cat#crl-3216); peripheral blood macrophages (human, Procell Science&Technology Cat#CP-H264)
Authentication	Cell identities were confirmed routinely by western blot and immunoflourescence marker expressions.
Mycoplasma contamination	Cell lines were routinely tested for mycoplasma contamination by mycoplasma detection kit and confirmed that they were negative for mycoplasma contamination.
Commonly misidentified lines (See ICLAC register)	No commonly misidentified cell lines were used.

Animals and other research organisms

Policy information about studies involving animals; ARRIVE guidelines recommended for reporting animal research, and Sex and Gender in Research

Laboratory animals	The 10-week old male or female ApoE-/- mice were purchased from Beijing Huafukang Bioscience Co., Ltd, China. The 10-12 week-old male C57BL/6 mice were purchased from Beijing Vital River Laboratory Animal Technology Co., Ltd, China. Mice were bred and housed under a Specific Pathogen-Free (SPF) status at a constant temperature (22-24°C) and humidity (50-70%) , under a 12 h-12 h light–dark cycle and provided with standard chow diet and water ad libitum. All mice were housed in static autoclaved HEPA-ventilated microisolator cages (27×16.5×15.5 cm) with autoclaved Enrich-o'Cobs (The Andersons Incorporated) for bedding. Cages and bedding were changed.
Wild animals	No wild animals were used in this study.
Reporting on sex	The FAM3A functions were detected respectively in male AAA mice and female AAA mice. The underlying mechanisms were analyzed in male mice. Epidemiologically, the AAA is a disease with a much higher incidence among male than female people.
Field-collected samples	No field-collected samples were used in this study.
Ethics oversight	All animal experiments were approved by the Ethics Review Board of Peking Union Medical College Hospital (ID: JS-2629).

Note that full information on the approval of the study protocol must also be provided in the manuscript.

Flow Cytometry

Plots

Confirm that:

☒ The axis labels state the marker and fluorochrome used (e.g. CD4-FITC).

☒ The axis scales are clearly visible. Include numbers along axes only for bottom left plot of group (a 'group' is an analysis of identical markers).

☒ All plots are contour plots with outliers or pseudocolor plots.

☒ A numerical value for number of cells or percentage (with statistics) is provided.

Methodology

Sample preparation	Mice were anesthetized using an intraperitoneal injection of sodium pentobarbital (45 mg/kg). The normal aorta (the supra-renal part of aorta without aneuysm from one mouse) or aneurysm tissue (the supra-renal part of aorta with aneurysm from one AAA mouse) were dissected and cut on ice, and digested for 15 min at 37°C in 1 mL PBS containing 50 U/ml collagenase I, 40 U/mL collagenase type XI, 40 U/mL hyaluronidase type I-s, 0.2 U/ml elastase, 5 U/ml neutral protease, and 0.3 U/ml deoxyribonuclease I. The digestion is terminated by PBS containing 2% fetal bovine serum, while the process can be repeated several times with the same amount of digestive enzymes until the tissue is completely digested. Cell suspensions were prepared by filtering through a cell strainer. The harvested cells were first stained with PE/Cy5.5-anti-CD34 and PB450-anti-

	CD68 for 30 minutes and washed with PBS. Then, the cells were fixed, permeabilized, and stained with APC-anti-αSMA, PE-anti-RUNX2 or LUM and PE/Cy5.5-anti-ADIPQ (different antibodies coupled by the same fluorescent group in a single flow assay were not used together).
Instrument	CytoFLEX (BeckmanCoulter, Inc, Bria, CA).
Software	CytExpert2.4.0.28 (Beckman Coulter, Inc, Bria, CA).
Cell population abundance	The number of cell samples obtained from each aortic tissue ranged from 1×10^4 to 20×10^4. According to the results of previous flow cytometry and single-cell RNA sequencing studies on Murine model aorta, the main cell populations in the cell samples included smooth muscle cells, endothelial cells, fibroblasts and macrophages .etc. In this study, only the changes of VSMCs were concerned, and flow cytometry results showed that the proportion of VSMCs after processing was about 5%-15%.
Gating strategy	For each sample of aortic tissue processed, the loading capacity of flow cytometry was between 10,000 and 20,000 cells. After cells clustered or with severely morphological changes were removed by grouping through forward scatter (FSC) and side scatter (SSC), the remaining cells were first gated by α-SMA. The cells selected as αSMA+ were then gated by the transdifferentiation marker (CD68 for example) to screen out the transdifferentiated VSMCs.

☒ Tick this box to confirm that a figure exemplifying the gating strategy is provided in the Supplementary Information.
